# Surgically assisted rapid palatal expansion using customized bone-borne devices

**DOI:** 10.4317/jced.54827

**Published:** 2018-07-01

**Authors:** Arturo Bilbao, Juan-Carlos Pérez-Varela, Daniel Pérez-López, Pablo Varela-Centelles

**Affiliations:** 1 Department of Surgery and Medical-Surgical Specialities. School of Medicine and Dentistry. University of Santiago de Compostela. Entrerríos s/n. 15782 Santiago de Compostela (A Coruña). Spain

## Abstract

Correction of maxillary compression via palatal expansion is easy in children and adolescents, but more complicated once growth is finished. This correction may be performed by progressive expansion using orthopaedic appliances after osteotomy with more stable results, which facilitate a second phase to achieve larger expansions. We present a clinical case treated using a customized device that improves predictability. The stability of the device is ensured by multiple support points with 8 screws that fix it to the palate.

** Key words:**Surgically assisted rapid palatal expansion, stereolithographic model, customized, bone-borne, expansion device.

## Introduction

Correction of maxillary compression via palatal expansion (1) is easy in children and adolescents, but more complicated once growth is finished with difficulties like remission, need for overcorrection, periodontal membrane compression, lateral dental displacement, or dental extrusion.

This correction may be performed using conventional orthognathic surgery (segmental LeFort I-type osteotomy), or progressive expansion using orthopaedic appliances after osteotomy (2) with more stable results, particularly for expansions >7 mm (3). This less aggressive approach offers less complications and facilitates a second phase to achieve larger expansions.

No spontaneous class III skeletal corrections occur following surgically-assisted expansion (4) and the use of elastics over mini-plates for traction produces good results when the anterior-posterior discrepancy is slight or moderate.

The osteotomy features and the need for pterygoid disjunction have been discussed (5). Despite this disjunction is widely accepted (6), the use of tooth- or bone-borne expansion devices remains controversial. Bone-borne devices provide more symmetric, greater overall expansion with less vestibular bone resorption and vestibulization of the teeth with a lower tilt of the fragments (7).

We tried a series of expansion devices, but the high cost and lack of stability prompted the modification of a Hyrax-type expansion device that could be screwed to the palatal bone. However, the individual variations in palatal fibromucosa thickness produced complications like decubitus at the device supporting points, impossibility of appropriately completing the treatment because of short screws in the device, and the difficulty of adjusting the mini-plates to the morphology of the palatal vault.

## Case Report

**Figure 1:**
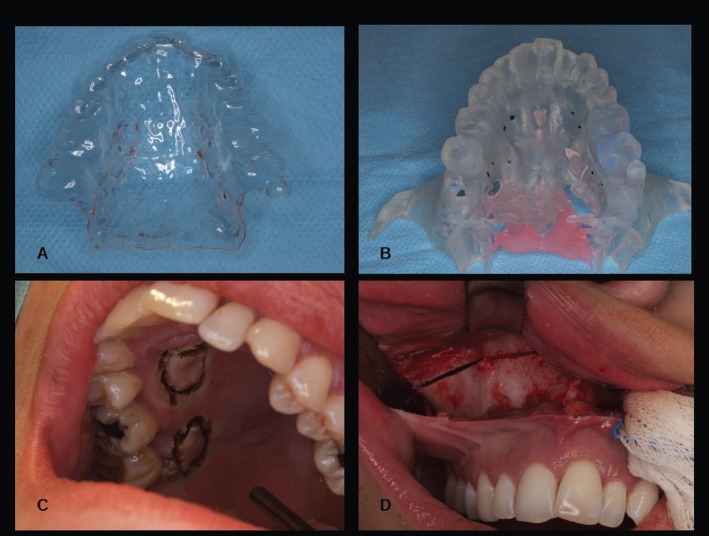
A: Mucous model; B: Stereolithographic bony model; C: Palatal incisions; D: LeFort I osteotomy.

After informed consent, an impression was taken and sealed with plaster, and a vacuum plate used to obtain a replica of the external aspect of the jaw (Fig. 1A).

A three-dimensional CT was used to build a stereolithographic model (Fig. 1B) reflecting the morphology of the jaw.

The 8 screws sites were marked in the model and the device manufactured from the welding of the steel mini-plate fragments over a Hyrax-type screw. The vacuum plate was placed over the model providing a space for the patient’s palatal fibromucosa.

The four rectangles in the plate corresponding to the marks from the model were cut, the location of the incisions for the device obtained, and the bone model adjusted to the individual morphology of the jaw.

The intervention was undertaken under local anaesthesia with midazolam intravenous sedation. Fentanyl was administered for analgesia and dexamethasone as anti-inflammatory. Antibiotic prophylaxis was performed using amoxicillin/clavulanic acid. The patient remained under outpatient care for an average of 3 hours.

We performed both vestibular (8) and vertical incisions in the midline and premolar regions for greater post-operative comfort with a diode laser and an electric scalpel to perform the palatal incisions. The fibromucosal rectangle corresponding to the splint of the incision was removed (Fig. 1C).

We set the expansion device and the self-drilling screws, which measured 2mm in diameter and 6mm in length.

**Figure 2:**
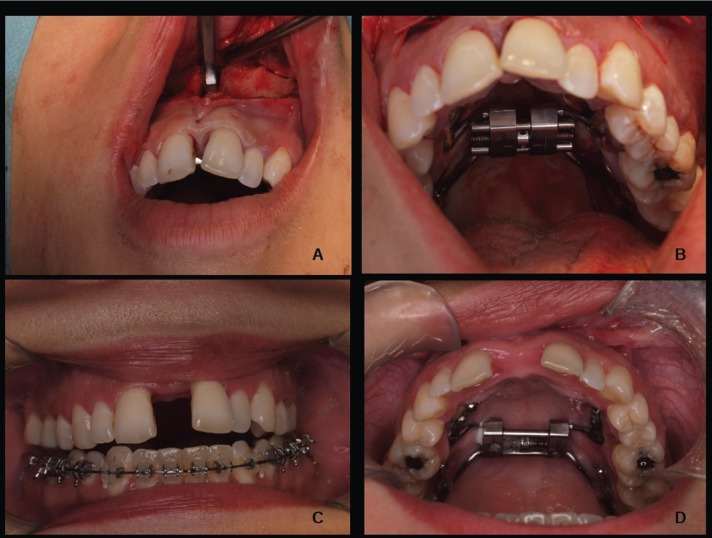
A: Sagittal osteotomy; B: End of surgery.; C: End of expansion. Front view; D: End of expansion. Palatal view.

Piezoelectric appliances were used to perform a complete, LeFort I-type osteotomy along the midline with movement of the jaw and pterygoid disjunction (9) (Fig. 1D,2A).

We activated the expansion device leaving a small gap for the haematoma constitution to form a bone repair callus that is modified during the expansion process (Fig. 2B).

We completed the surgery using a high molecular weight hyaluronic acid gel over the flaps and edges of the wound to improve healing and reduce the incidence of retractile scars and increase patient comfort.

We continued the antibiotic regimen of amoxicillin/clavulanic acid 875/125 mg every 8 hours for 8 days and ibuprofen 600 mg every 8 hours for 8 days during the post-operative period.

The protocol for other craniofacial sites was also followed in this study because this approach also involves distraction osteogenesis: the allowed latency period was 5 days, with an expansion speed of 0.5-1 mm/day and a consolidation period of 6 to 12 months (10).

The diastema closed 8 weeks after the expansion, as marked by the formation of a bone callus in the distraction chamber. The expansion device was removed 90 days after the closing of the diastema (Fig. 2C,D).

Palatal expansion displays several advantages over segmental LeFort I-type osteotomy (6), including less aggressiveness, greater stability, and greater patient acceptance. We also add the possibility of the application of new technologies to create a customized device from a faithful reproduction of the patient’s bone anatomy.

## Discussion

Previous devices have been manufactured using a traditional impression with the inclusion of the mucosal component or standardized measurements that minimize the possibilities for adjustment based on hinging mechanisms. Our device produces a more predictable and stable expansion that is reinforced by multiple support points at the 8 screws that are used in the setting of the expansion device.

Future multicenter and comparative studies will provide a better understanding of the differences in and advantages of the use of these devices.
